# A Single Conserved Amino Acid Residue as a Critical Context-Specific Determinant of the Differential Ability of Mdm2 and MdmX RING Domains to Dimerize

**DOI:** 10.3389/fphys.2019.00390

**Published:** 2019-04-09

**Authors:** Pavlína Kosztyu, Iva Slaninová, Barbora Valčíková, Amandine Verlande, Petr Müller, Jan J. Paleček, Stjepan Uldrijan

**Affiliations:** ^1^ Department of Biology, Faculty of Medicine, Masaryk University, Brno, Czechia; ^2^ International Clinical Research Center, St. Anne’s University Hospital, Brno, Czechia; ^3^ Regional Centre for Applied Molecular Oncology, Masaryk Memorial Cancer Institute, Brno, Czechia; ^4^ Central European Institute of Technology, Masaryk University, Brno, Czechia; ^5^ National Centre for Biomolecular Research, Faculty of Science, Masaryk University, Brno, Czechia

**Keywords:** Mdm2, Mdm4, MdmX, RING domain ubiquitin protein ligase, dimerization, mutagenesis, E3, p53

## Abstract

Mdm2 and MdmX are related proteins serving in the form of the Mdm2 homodimer or Mdm2/MdmX heterodimer as an E3 ubiquitin ligase for the tumor suppressor p53. The dimerization is required for the E3 activity and is mediated by the conserved RING domains present in both proteins, but only the RING domain of Mdm2 can form homodimers efficiently. We performed a systematic mutational analysis of human Mdm2, exchanging parts of the RING with the corresponding MdmX sequence, to identify the molecular determinants of this difference. Mdm2 can also promote MdmX degradation, and we identified several mutations blocking it. They were located mainly at the Mdm2/E2 interface and did not disrupt the MdmX-Mdm2 interaction. Surprisingly, some mutations of the Mdm2/E2 interface inhibited MdmX degradation, which is mediated by the Mdm2/MdmX heterodimer, but did not affect p53 degradation, mediated by the Mdm2 homodimer. Only one mutant, replacing a conserved cysteine 449 with asparagine (C449N), disrupted the ability of Mdm2 to dimerize with MdmX. When we introduced the cysteine residue into the corresponding site in MdmX, the RING domain became capable of forming dimers with other MdmX molecules *in vivo*, suggesting that one conserved amino acid residue in the RINGs of Mdm2 and MdmX could serve as the determinant of the differential ability of these domains to form dimers and their E3 activity. In immunoprecipitations, however, the homodimerization of MdmX could be observed only when the asparagine residue was replaced with cysteine in both RINGs. This result suggested that heterocomplexes consisting of one mutated MdmX RING with cysteine and one wild-type MdmX RING with asparagine might be less stable, despite being readily detectable in the cell-based assay. Moreover, Mdm2 C449N blocked Mdm2-MdmX heterodimerization but did not disrupt the ability of Mdm2 homodimer to promote p53 degradation, suggesting that the effect of the conserved cysteine and asparagine residues on dimerization was context-specific. Collectively, our results indicate that the effects of individual exchanges of conserved residues between Mdm2 and MdmX RING domains might be context-specific, supporting the hypothesis that Mdm2 RING homodimers and Mdm2-MdmX heterodimers may not be entirely structurally equivalent, despite their apparent similarity.

## Introduction

Mdm2 and MdmX (also known as Mdm4) are closely related proteins which work together to control the levels and activity of the tumor suppressor p53 during embryonic development and in unstressed healthy cells (Jones et al., 1995; Montes de Oca Luna et al., 1995; Parant et al., 2001; Migliorini et al., 2002; Marine et al., 2006; Ringshausen et al., 2006). However, in human cancers retaining wild-type *p53* gene, Mdm2 and MdmX proteins are often expressed at high levels, overcoming the growth-suppressive functions of p53 and contributing to tumor development (Momand et al., 1998; Toledo and Wahl, 2006). Mdm2 and MdmX can directly interact with p53 and inhibit its transcription activity (Momand et al., 1992; Chen et al., 1993; Shvarts et al., 1996). Both proteins also serve as RING finger E3 ubiquitin ligases for p53, either in the form of an Mdm2 homodimer or Mdm2-MdmX heterodimer (Fang et al., 2000; Uldrijan et al., 2007). Mdm2 can also serve as E3 for MdmX and for Mdm2 itself. In the absence of MdmX, Mdm2 is unstable and less active toward p53. Dimerization with MdmX increases its stability and E3 activity toward p53. At the same time, the interaction with Mdm2 promotes the translocation of MdmX from the cytoplasm to the nucleus where the wild-type p53 protein predominantly resides (Stad et al., 2001; Gu et al., 2002; Li et al., 2002).

**Figure 1:**
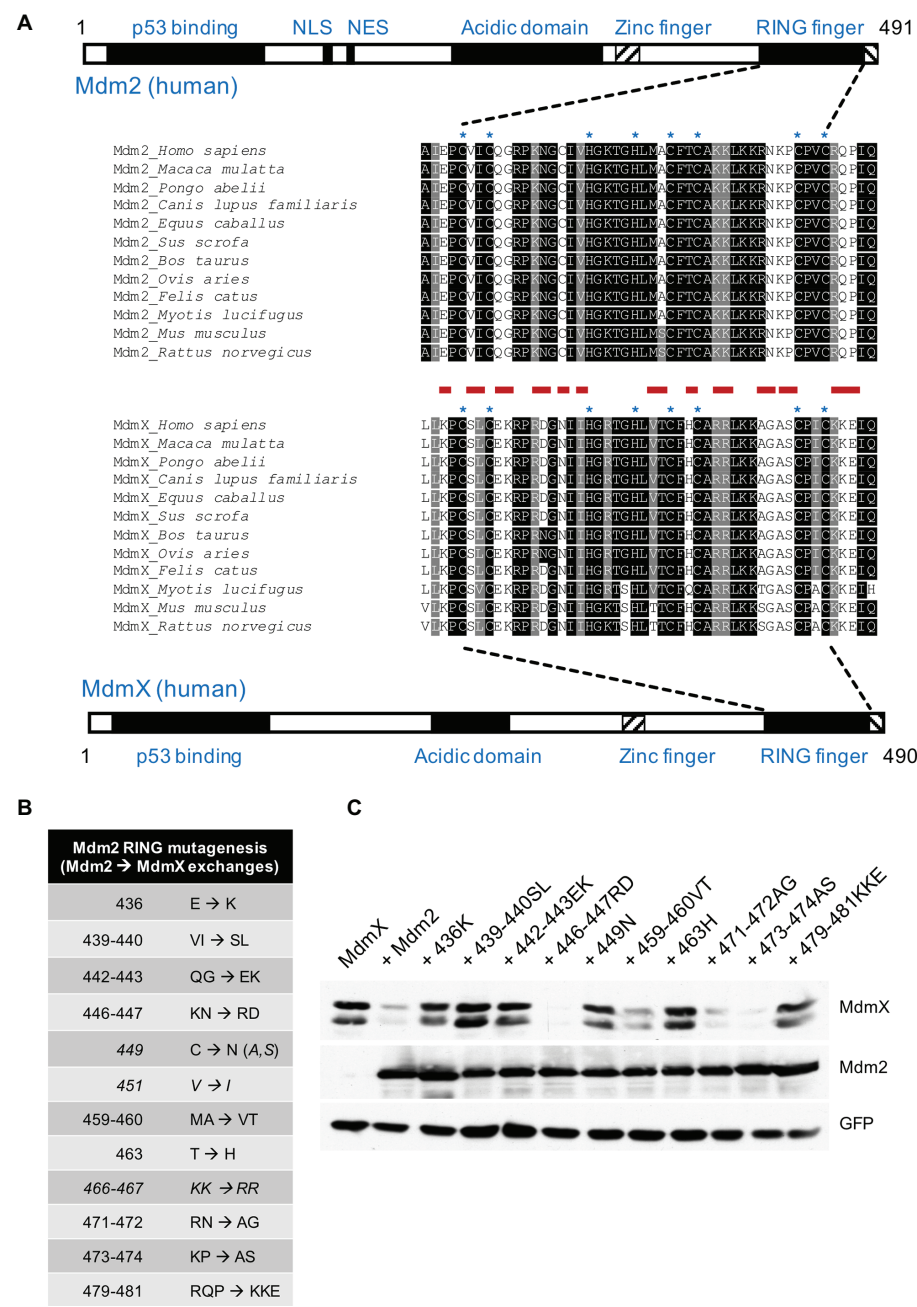
Mutations mimicking MdmX RING sequence inhibit Mdm2 activity toward MdmX. **(A)** Schematic representation of human Mdm2 and MdmX proteins. RING domain sequences of selected mammalian Mdm2 and MdmX proteins were aligned using BOXSHADE 3.21 software at http://www.ch.embnet.org/software/BOX_form.html. Zinc coordinating residues are marked with an asterisk (*). Sites individually mutated in this study are marked in red. **(B)** Mdm2 mutants generated for this study. Selected amino acid residues were replaced with the corresponding MdmX RING residues. Some mutants presented in the table were created at later stages of the project (shown in *italics*). **(C)** The activity of selected mutants was tested in the MdmX degradation assay. U2OS cells were transiently transfected with combinations of plasmid vectors coding for Myc-tagged MdmX, GFP, and wild-type Mdm2 or the RING domain Mdm2 mutants. Lysates of transfected cells were analyzed by Western blotting.

The N-terminal p53 binding domains and the RING domains of Mdm2 and MdmX are highly conserved in evolution (Figure 1A), as is the central acidic domain of Mdm2, which also actively participates in p53 ubiquitination and degradation (Argentini et al., 2001; Kawai et al., 2003; Meulmeester et al., 2003; Dolezelova et al., 2012b; Tan et al., 2016). The RING domains are in both proteins located very close to the C-terminus (Figure 1A) and the adjacent C-terminal tails, conserved in length and sequence, also participate in the RING domain function (Poyurovsky et al., 2007; Uldrijan et al., 2007). Although the structures of the two RING domains are very similar (Kostic et al., 2006; Linke et al., 2008), the Mdm2 homodimers and Mdm2/MdmX heterodimers do not seem to be structurally and functionally fully equivalent (Dolezelova et al., 2012a). The MdmX RING finger does not possess the ubiquitin ligase activity toward p53 on its own but can stimulate the Mdm2-mediated p53 ubiquitination and restore the E3 activity of Mdm2 mutants disrupting the function of the C-terminal tail (Linares et al., 2003; Uldrijan et al., 2007). This difference between MdmX and Mdm2 could be in part caused by intramolecular interactions mediated by other regions of the proteins. MdmX has been reported to contain autoinhibitory sequence elements that compete with the binding of MdmX to the transactivation domain of p53 (Bista et al., 2013; Chen et al., 2015). The RING domain of Mdm2 was shown to physically interact with the central acidic region of Mdm2 (Dang et al., 2002). The primary amino acid sequences of the central domains are less conserved between Mdm2 and MdmX, and MdmX cannot provide the critical function of the acidic domain in Mdm2-mediated p53 ubiquitination. A short sequence within the acidic domain of Mdm2 has been identified as necessary for p53 ubiquitination and a 30-amino acid region encompassing this sequence has been shown to promote the ubiquitin ligase activity of Mdm2 by an intramolecular interaction with the RING domain (Dolezelova et al., 2012b; Cheng et al., 2014).

A notable feature of RING-type E3s is their tendency to form homodimers and heterodimers that seems to be the prerequisite for the E3 activity for many RING finger ubiquitin ligases (Metzger et al., 2014). The RING domain of Mdm2 is capable of forming both homodimers and heterodimers with MdmX RING, while the RING domain of MdmX does not homodimerize and can form active ubiquitin ligase only in the form of MdmX-Mdm2 heterodimer (Tanimura et al., 1999; Kawai et al., 2007). MdmX stabilizes Mdm2 and stimulates Mdm2-mediated p53 ubiquitination and degradation (Sharp et al., 1999; Stad et al., 2000; Gu et al., 2002; Linares et al., 2003). Hetero-oligomerization with MdmX rescues the ubiquitin ligase activity of Mdm2 C-terminal mutants (Poyurovsky et al., 2007; Singh et al., 2007; Dolezelova et al., 2012a). Moreover, the Mdm2/MdmX heterocomplex seems to be the predominant form present in cells, required for the control of p53 activity *in vivo* (Kawai et al., 2007; Huang et al., 2011). A precise understanding of the nature of Mdm2-MdmX interactions can be critical to exploiting them as potential therapeutic targets for reactivation of p53 function in tumors. In this study, we systematically analyzed the primary structure of Mdm2 and MdmX RING domains to identify critical differences that render the MdmX RING inactive by preventing its dimerization and E3 activity.

## Materials and Methods

### Cell Culture

Human U2OS and HEK293 cell lines were obtained from ECACC and DSMZ, respectively. Cells were cultured at 37°C / 5% CO_2_ in Dulbecco’s modified Eagle’s medium (DMEM) supplemented with 10% fetal calf serum, 2 mM L-glutamine, 50 U/ml penicillin G, and 50 μg/ml streptomycin sulfate (all from Sigma-Aldrich).

### Plasmids and Mutagenesis

Mammalian expression plasmids coding for wild-type Mdm2, Mdm2 mutant Mdm2∆9, lacking the conserved C-terminal tail required for RING dimerization, Mdm2 mutant C464A, disrupting the RING domain structure and function, Myc-tagged MdmX, FLAG-tagged human wild-type p53 (pcDNA3-FLAG-p53), and the Mdm2/MdmX chimera (MDM2/MDMXRFD) have been described previously (Chen et al., 1993; Sharp et al., 1999; Kawai et al., 2003; Uldrijan et al., 2007). GFP-MdmX expression plasmid coding for N-terminally tagged full-length human MdmX was kindly provided by Robert Ludwig (The Beatson Institute, Glasgow, UK). Plasmid pRK5-HA-Ubiquitin-WT encoding hemagglutinin-tagged ubiquitin was a gift from Ted Dawson (Addgene plasmid # 17608). Empty vectors pcDNA3.1 and pEGFP-N1 were obtained from Invitrogen and Clontech, respectively. All mutations within the RING domains of Mdm2 and MdmX were generated by PCR-mediated site-directed mutagenesis using Pfu DNA polymerase (Stratagene) and verified by DNA sequencing.

### p53 Degradation Assay

U2OS cells in 60-mm dishes were transfected with 0.1 μg FLAG-p53 and 1.4 μg Mdm2 mutant plasmid constructs using Effectene transfection reagent according to guidelines of the manufacturer (Qiagen). Each transfection mixture also contained 0.2 μg pEGFP-N1 (Clontech) to control for transfection efficiency, and empty plasmid pcDNA3.1 (Invitrogen) was used to bring the total amount of transfected DNA to 1.7 μg. Cells were washed with cold PBS 30 h post-transfection and lysed with 0.25 ml of SDS sample buffer. Proteins were resolved by SDS-polyacrylamide gel electrophoresis (SDS-PAGE) and analyzed by Western blotting with anti-Flag M2 (Sigma-Aldrich, F3165, 1:3,000), anti-Mdm2 Ab-1 (Merck, OP46, 1:1,000), and anti-GFP 7.1/13.1 (Roche, 11814460, 1:5,000) antibodies. In some experiments, p53 was also detected using the anti-p53 monoclonal antibody DO-1 (used at 0.2 μg/ml, kindly provided by Borivoj Vojtesek, Masaryk Memorial Cancer Institute, Brno).

### MdmX Degradation Assay

U2OS cells grown in 60-mm dishes were transfected with 1 μg Myc-MdmX and 6 μg wild-type Mdm2 or Mdm2 mutant plasmid constructs using Lipofectamine 2000 transfection reagent (Invitrogen) and DMEM was changed 4 h later. Each transfection mixture also contained 0.1 μg pEGFP-N1 as a control for transfection efficiency. The empty plasmid pcDNA3.1 was used to bring the total amount of transfected DNA to 7.1 μg in cells transfected with MdmX expression plasmid alone. Cells were cultured for 30 h, washed with cold PBS, and lysed with 0.3 ml of SDS-PAGE sample buffer. Proteins were resolved by SDS-PAGE and analyzed by Western blotting with anti-Myc 9E10 (Merck, MABE282, 1:1,000), anti-Mdm2 Ab-1 (Merck, OP46, 1:1,000), and anti-GFP 7.1/13.1 (Roche, 11814460, 1:5,000) antibodies.

### MdmX Relocalization Assay

U2OS cells grown on coverslips were transfected with Myc-tagged MdmX or GFP-tagged MdmX (0.3 μg), and wild-type Mdm2 expression plasmids or the Mdm2/MdmX chimeric construct (1.2 μg) using Effectene transfection reagent (Qiagen) and the culture medium was changed 4 h later. The empty plasmid pcDNA3.1 was used to bring the total amount of transfected DNA to 1.5 μg in cells transfected with MdmX expression plasmid alone. Twenty-four hours post-transfection, cells were treated with 15 μM MG132 (Sigma-Aldrich) in DMEM for 3 h, washed with PBS, and fixed in 4% paraformaldehyde in PBS for 10 min at room temperature. After fixation, cells were washed with PBS and permeabilized with PBS containing 0.2% Triton X-100 (Sigma-Aldrich) for 5 min. Cells were blocked with PBS containing 0.5% bovine serum albumin at room temperature for 30 min and then incubated for 2 h at room temperature with anti-Mdm2 mouse monoclonal antibody IF2 (Ab-1, Merck, OP46, 1:200) in the blocking solution or the anti-Mdm2 antibody together with anti-c-Myc rabbit polyclonal antibody (A-14, Santa Cruz Biotechnology sc-789, 1:250). Cells were washed with PBS and incubated for 1 h at room temperature with FITC- or DyLight^™^594-conjugated secondary antibodies (Jackson ImmunoResearch) in the blocking solution containing 1 μg/ml DAPI (Sigma-Aldrich). Cells were washed with PBS and mounted with VECTASHIELD HardSet Antifade Mounting Medium (Vector Laboratories). Images were taken with the FluoView 500 confocal laser scanning fluorescence microscopes (Olympus).

### Immunoprecipitations

HEK293 cells were transfected with 6 μg DNA per 100-mm plate using Lipofectamine 2000 reagent (Invitrogen). Cells were treated with a proteasome inhibitor (10 μM MG132 in cell culture medium) 24–36 h post-transfection, 4 h later washed with PBS, and lysed in Triton X-100 lysis buffer (1% Triton X-100, 150 mM NaCl, and 50 mM Tris pH 8.0) containing protease inhibitors (Complete Mini, Roche). Lysates were pre-cleared with 50 μl of protein G-Sepharose (Millipore). Immunoprecipitations were performed at 4°C with 1–2 μg of an antibody bound to 50 μl of protein G-Sepharose for 1 h. Mdm2 was immunoprecipitated with anti-Mdm2 antibody IF2 (Ab-1, Merck, OP46) and Myc-MdmX with anti-Myc antibody 9E10 (Merck, MABE282). Immunoprecipitated proteins were washed with lysis buffer and resuspended in SDS-PAGE sample buffer. Proteins from whole-cell extracts and immunoprecipitations were resolved by SDS-PAGE and analyzed by Western blotting with anti-Mdm2 Ab-1 (Merck, OP46, 1:1,000) and anti-Myc 9E10 (Merck, MABE282, 1:1,000) or anti-Myc A-14 (Santa Cruz Biotechnology, sc-789, 1:1,000).

### Mdm2-Mediated p53 Ubiquitylation in Cells

U2OS cells grown in 60-mm dishes were transiently transfected with FLAG-p53 (0.5 μg), hemagglutinin (HA)-ubiquitin (0.5 μg), and Mdm2 plasmid constructs (5 μg) using Lipofectamine 2000 reagent (Invitrogen) according to the manufacturer’s recommendations. Empty plasmid pcDNA3.1 (Invitrogen) was used to bring the total amount of transfected DNA to 6 μg. Cells were treated 24 h post-transfection with 15 μM MG132 for 3 h, washed with PBS, and lysed in 0.3 ml 0.5% SDS. Lysates were boiled for 5 min, vortexed, cooled down to room temperature, and diluted with 1 ml of Triton X-100 lysis buffer. p53 was immunoprecipitated using 1 μg of anti-p53 monoclonal antibody DO-1 on a rotating wheel at 4°C. After 1 h of incubation, 20 μl of protein G-Sepharose beads (Sigma-Aldrich) were added for 45 min. The immunoprecipitates were washed three times with lysis buffer and analyzed by Western blotting. Ubiquitylated p53 was detected using anti-HA-Peroxidase antibody (Roche, 12013819001, 1:2,000); FLAG-p53 and MDM2 levels in the input were detected using anti-FLAG antibody M2 (Sigma-Aldrich), anti-Mdm2 Ab-1 (Merck).

### Modeling the MdmX-Mdm2-E2 Interactions

The Mdm2 contact residues mediating its binding to the MdmX and UbcH5b partners have been identified using COZOID (COntact ZOne IDentifier) tool (Furmanová et al., 2018; http://decibel.fi.muni.cz/cozoid/). The 2VJF, 2HDP, and 5MNJ structural data have been used for the Mdm2/MdmX analysis and visualization. Structures were visualized and simulated using the PyMOL tool (Molecular Graphics System, Version 1.7.2, Schrodinger, LLC; http://www.pymol.org).

## Results

### The Systematic Mutational Analysis Identifies Mdm2 RING Residues Participating in MdmX Degradation

To identify conserved regions that could be responsible for the differential ability of Mdm2 and MdmX to form homodimers, we performed multiple alignments of Mdm2 and MdmX RING domain primary amino acid sequences of various mammalian species (Figure 1A). PCR-mediated site-directed mutagenesis was then used to introduce amino acid changes in the RING domain of full-length human Mdm2. In each of the mutants, a tiny part of the Mdm2 sequence (one, two, or three amino acids) was exchanged with the amino acid residues present in the corresponding region of human MdmX RING domain (Figure 1B). A cell-based MdmX degradation assay showed that many of the mutations introduced into Mdm2 RING disrupted the ability to target MdmX for degradation (Figure 1C). More specifically, the mutants 436K, 439–440SL, 442–443EK, 449N, 463H, and 479–481KKE had significantly impaired capacity to promote MdmX degradation, indicating that multiple differences between Mdm2 and MdmX could be responsible for the lack of E3 activity in MdmX RING and possibly also for its inability to form dimers. Therefore, in the next set of experiments, we analyzed the potential impact of the mutations on the physical interaction of the Mdm2 RING with the MdmX RING domain.

### A Single MdmX Residue Disrupts the Ability of Mdm2 RING to Bind MdmX

As already mentioned, Mdm2 not only regulates the stability of MdmX but can also induce changes in the subcellular localization of MdmX and promote its nuclear accumulation (Stad et al., 2001; Li et al., 2002). Mdm2 and MdmX proteins differ in the presence of signals for subcellular localization (Figure 1A). MdmX lacks the nuclear localization signal (NLS) and the nuclear export signal (NES) and is predominantly located in the cytoplasm, while Mdm2 contains both signals and shuttles continuously between the cell nucleus and cytoplasm. Despite this ability to dynamically change subcellular location, the majority of ectopically expressed Mdm2 protein can be detected in the nucleus of U2OS osteosarcoma cells. Importantly, when wild-type Mdm2 and MdmX are co-expressed at approximately 1:1 ratio, they dimerize through their respective RING domains, and the Mdm2-MdmX heterodimer localizes mainly to cell nucleus (Uldrijan et al., 2007). We performed this relocalization assay with all the Mdm2–›MdmX RING domain mutants. Additional mutants 451I and 466-467RR were generated at this stage and included in the relocalization experiment to more thoroughly cover the differences between Mdm2 and MdmX RING domains.

**Figure 2:**
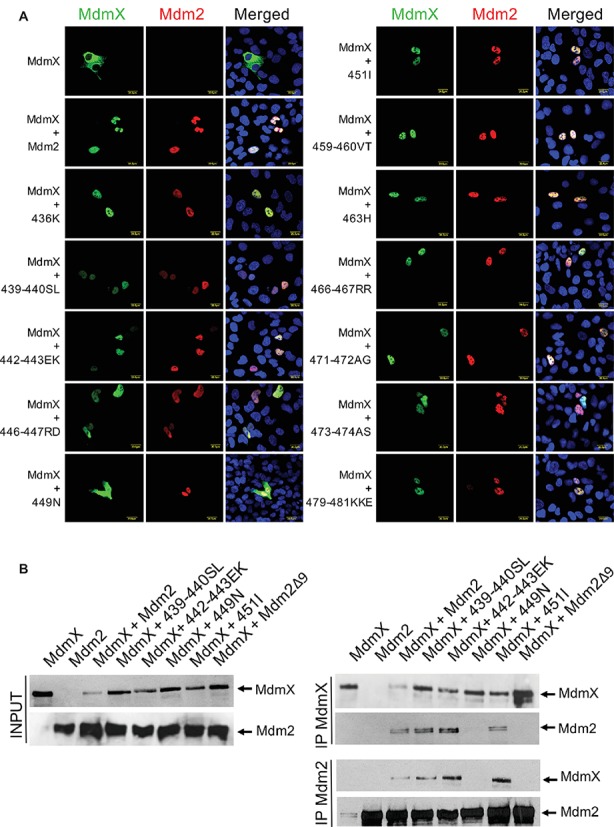
Most Mdm2 mutants mimicking MdmX RING sequence retain the ability to heterodimerize with MdmX. **(A)** MdmX relocalization assay. U2OS cells were transfected with GFP-tagged MdmX expression plasmid together with plasmids coding for wild-type Mdm2 or the Mdm2 RING mutants. MdmX was detected by GFP fluorescence (green) and Mdm2 by immunofluorescence (red). DAPI was used to label cell nuclei (blue). **(B)** Immunoprecipitations. Plasmid constructs encoding selected Mdm2 mutants were transiently transfected into HEK293 cells together with Myc-tagged MdmX plasmid construct. Cells were treated 24 h post-transfection with the proteasome inhibitor MG132 for 4 h, lysed, and immunoprecipitated with anti-Mdm2 and anti-Myc antibodies. Immunoprecipitates were analyzed by Western blotting. INPUT: Mdm2 and MdmX levels in cell lysates. IP MdmX: Mdm2 and MdmX levels in samples immunoprecipitated using the anti-Myc tag antibody. IP Mdm2: MdmX and Mdm2 levels in samples immunoprecipitated with the anti-Mdm2 antibody.

To our surprise, the results suggested that all but one could physically interact with MdmX in living cells (Figure 2A). The only exception was the 449N mutant in which a single cysteine residue at position 449 of Mdm2 (C449) was exchanged with asparagine that is present at the corresponding position of MdmX RING (N448). The defect in MdmX interaction of the 449N Mdm2 mutant was confirmed in immunoprecipitations (Figure 2B). Wild-type Mdm2 and the 439–440SL and 442–443EK mutants that did not induce MdmX degradation but did not show a defect in the MdmX relocalization assay were used as positive controls, while the Mdm2∆9 mutant lacking the conserved C-terminal tail required for Mdm2 RING dimerization was used as a negative control.

**Figure 3:**
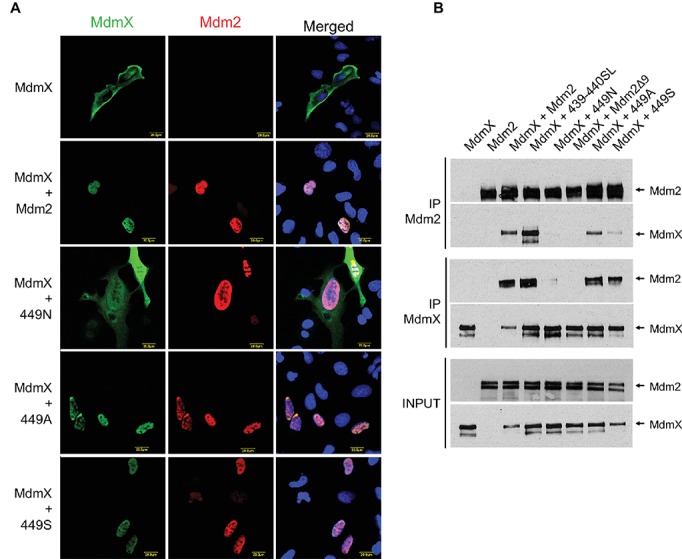
C449N mutation disrupts Mdm2-MdmX heterodimerization and MdmX degradation. **(A)** MdmX relocalization assay. U2OS cells were transfected with GFP-tagged MdmX together with wild-type Mdm2 or selected Mdm2 C449 mutants. MdmX was detected by GFP fluorescence (green) and Mdm2 by immunofluorescence (red). DAPI was used to label cell nuclei (blue). **(B)** Immunoprecipitations. Selected Mdm2 mutants were transiently transfected into HEK293 cells together with Myc-tagged MdmX. Cells were treated 24 h post-transfection with the proteasome inhibitor MG132 for 4 h, lysed, and immunoprecipitated with anti-Mdm2 and anti-Myc antibodies. Immunoprecipitates were analyzed by Western blotting. INPUT: Mdm2 and MdmX levels in cell lysates. IP MdmX: Mdm2 and MdmX levels in samples immunoprecipitated using the anti-Myc tag antibody. IP Mdm2: MdmX and Mdm2 levels in samples immunoprecipitated with the anti-Mdm2 antibody.

Both C449 and N448 are conserved in the Mdm2 and MdmX RING domains of all analyzed mammalian species (Figure 1A). As they are situated at the Mdm2-MdmX RING-RING interface (Linke et al., 2008; Nomura et al., 2017), these specific residues might be required for the optimal regulation of Mdm2-MdmX interactions. To test this hypothesis, we created additional Mdm2 RING mutants in which C449 was exchanged with alanine (449A) and serine (449S) residues. Analyses of the ability of the new mutants to interact with MdmX in the relocalization assays (Figure 3A) and immunoprecipitations (Figure 3B) indicated that, unlike 449N, they retained the ability to bind MdmX. However, the results of the immunoprecipitations suggested that the interaction between MdmX and the 449S mutant might be weaker than the interaction with wild-type Mdm2. These results indicated that only some residues at position 449 might be optimal for Mdm2 activity toward MdmX, potentially contributing to the conservation of cysteine 449 of Mdm2 in evolution.

### Exchange of a Single Residue Stimulates MdmX RING Domain Homodimerization

**Figure 4:**
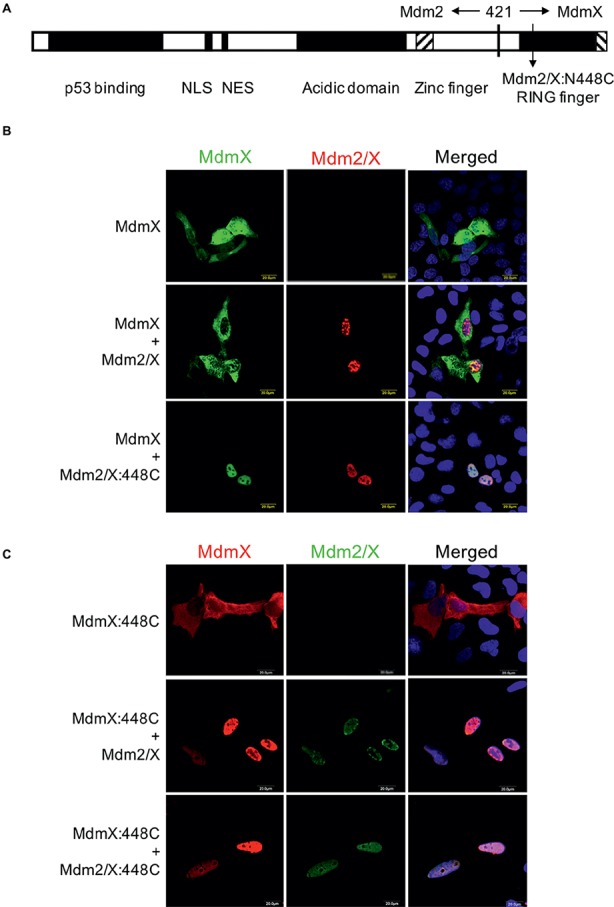
Replacing N448 of MdmX RING with cysteine to mimic Mdm2 C449 promotes MdmX homodimerization *in vivo*. **(A)** Schematic representation of the Mdm2/MdmX chimeric protein (Mdm2/X). **(B)** MdmX relocalization assay. U2OS cells were transfected with GFP-tagged MdmX plasmid construct together with plasmids coding for Mdm2/X or Mdm2/X:448C in which the critical asparagine residue was replaced with cysteine. MdmX was detected by GFP fluorescence (green) and Mdm2/X by immunofluorescence (red). DAPI was used to label cell nuclei (blue). **(C)** MdmX relocalization assay. U2OS cells were transfected with plasmid construct encoding Myc-MdmX, in which the critical asparagine residue was replaced with cysteine (MdmX:448C), together with plasmids coding for Mdm2/X or Mdm2/X:448C. MdmX (red) and Mdm2/X (green) were detected by immunofluorescence. DAPI was used to label cell nuclei (blue).

In the next set of experiments, we turned our attention to the RING domain of MdmX. We performed site-directed mutagenesis of the conserved asparagine 448 in MdmX RING into cysteine to mimic the critical C449 residue of Mdm2. However, to be able to test the effect of the mutation on MdmX homodimerization in the relocalization assay, we mutated N448 not only in the context of the full-length MdmX protein but also in a previously constructed Mdm2/MdmX chimera (Mdm2/X). This protein retains most of the Mdm2 sequence (including the NLS and NES localization signals) but the C-terminal portion of Mdm2 including the RING domain was exchanged with the MdmX sequence [Figure 4A, (Kawai et al., 2003)]. Like wild-type Mdm2, the Mdm2/X chimeric protein localizes mainly to the nucleus, but it is not able to relocalize wild-type MdmX into cell nucleus as two MdmX RING domains do not interact. However, when we introduced cysteine instead of asparagine at the position corresponding to N448 in the MdmX RING domain of the chimera (Mdm2/X:448C), it induced nuclear localization of the wild-type MdmX protein (Figure 4B). Analogically, when the cysteine residue was introduced into the MdmX protein (MdmX:448C), the unmutated Mdm2/X chimera, retaining asparagine at the position corresponding to 448 of MdmX, was able to relocalize the mutated MdmX protein to the nucleus (Figure 4C). This result confirmed the critical importance of the cysteine residue present in Mdm2 at position 449 for the RING domain-mediated dimerization. Moreover, it indicated that the ability of two MdmX RING domains to dimerize could be induced already by introducing the critical cysteine residue into one of the interacting RING domains, in a manner similar to the heterodimerization between Mdm2 and MdmX RING domains.

**Figure 5:**
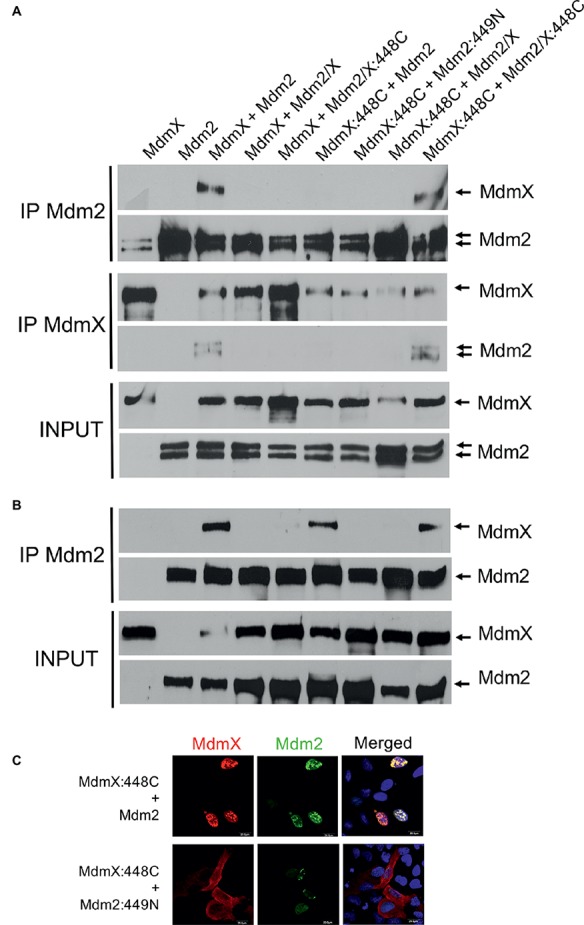
MdmX N448C RING domain dimerization **(A)** Immunoprecipitations. Mdm2, Myc-MdmX, and Mdm2/X plasmid constructs were transiently transfected into HEK293 cells. Cells were treated 24 h post-transfection with proteasome inhibitor MG132 for 4 h, lysed, and immunoprecipitated with an anti-Mdm2 antibody. Immunoprecipitates were analyzed by Western blotting. INPUT: Mdm2 and MdmX levels in cell lysates. IP MdmX: Mdm2 and MdmX levels in samples immunoprecipitated using the anti-Myc tag antibody. IP Mdm2: MdmX and Mdm2 levels in samples immunoprecipitated with the anti-Mdm2 antibody. MdmX RING homodimerization was observed only when both RINGs contained a cysteine residue at position 448. **(B)** Immunoprecipitations. In addition to the MdmX 448C-MdmX 448C homodimerization, the interaction between MdmX 448C and wild-type Mdm2 was also observed in some immunoprecipitation experiments. Samples were loaded in the same order as in **(A)**. **(C)** MdmX relocalization assay. The interaction between MdmX 448C (red) and Mdm2 (green) was observed also in this assay. Experiment performed as in Figure 4C.

Interestingly, in immunoprecipitations, the interaction of two MdmX RINGs was observed only when the cysteine residue was introduced into both of them (Figure 5A), suggesting that heterocomplexes containing one MdmX RING with cysteine at position 448 and one wild-type MdmX RING with arginine at the same position might be less stable. Interestingly, two out of three performed immunoprecipitation assays showed that MdmX 448C could also interact with wild-type Mdm2, but not with Mdm2 449N (Figure 5B). A similar difference was also seen in the cell-based relocalization assay (Figure 5C). This result suggested that we could not recapitulate the normal mode of Mdm2-MdmX interactions by merely swapping the critical C449/N448 residues between Mdm2 and MdmX. Therefore, it is probable that other residues in the context of the RING domains are also significantly contributing to the optimal Mdm2-MdmX dimerization.

### Differential Participation of the Conserved RING Residues in the Activity of Mdm2 Homodimers and Mdm2/MdmX Heterodimers

**Figure 6:**
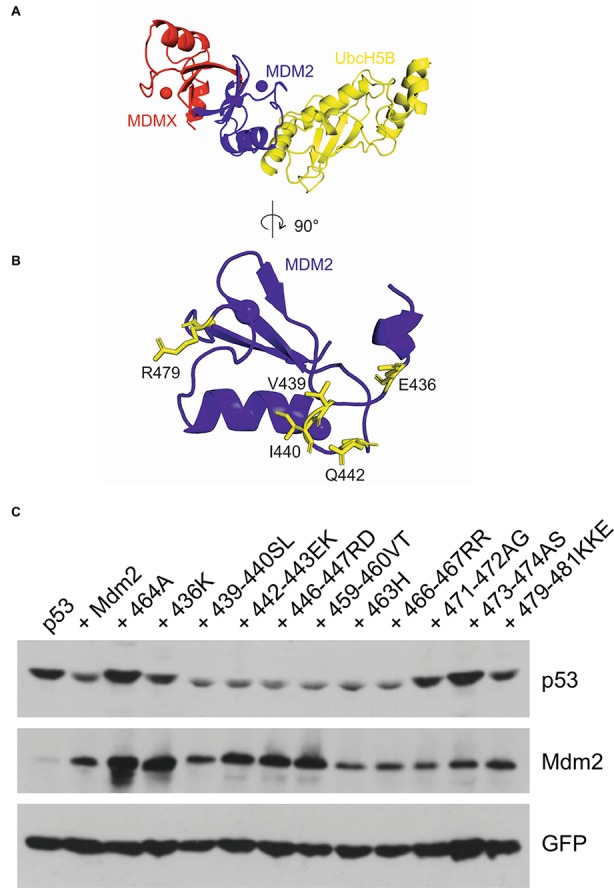
Mutations of Mdm2/E2 contact residues can have a different impact on Mdm2 activity toward different targets. **(A)** Structure of the MdmX-Mdm2-UbcH5b complex (PDB: 5MNJ) (Nomura et al., 2017). Mdm2 (blue) binds MdmX (red) and UbcH5b (yellow) *via* opposite contact surfaces. **(B)** The Mdm2 contact surface view with the E436, V439, I440, Q442, and R479 contact residues mediating Mdm2-UbcH5b interaction highlighted in yellow. (**C**) p53 degradation assay. U2OS cells were transfected with plasmids coding for GFP, FLAG-tagged p53, and wild-type Mdm2 or selected Mdm2 mutants. Cells were lysed 30 h post-transfection and proteins were resolved by SDS-PAGE and analyzed by Western blotting.

The structural analyses of Mdm2 homodimers and Mdm2-MdmX heterodimers indicated that Mdm2 RING binds both the E2 ubiquitin-conjugating enzyme and ubiquitin, while MdmX does not bind E2 and contacts only ubiquitin, *via* its C-terminal tail (Kostic et al., 2006; Linke et al., 2008; Nomura et al., 2017). We modeled the Mdm2/E2 interface using the recently published MdmX-Mdm2-E2 structure and found that potentially critical residues E436, V439, I440, Q442, and R479 were disrupted in the mutants that were inactive in the MdmX degradation assay (Figures 6A,B). Their location at the Mdm2/E2 interface could help to explain why the MdmX residues in 436K, 439–440SL, 442–443EK, and 479–481KKE inhibited the activity of these mutants toward MdmX. We reasoned that mutations actively disrupting the Mdm2/E2 interface and E2 binding should inhibit the E3 activity of not only Mdm2-MdmX heterodimers but also Mdm2 homodimers. To our surprise, however, in a p53 degradation assay, we observed a decreased activity toward p53 of mutants 436K and 479–481KKE, while mutants 439–440SL and 442-442EK remained fully active (Figure 6C). These results indicated that the contribution of individual residues at the Mdm2/E2 interface to E2 binding might differ in Mdm2 homodimers and Mdm2-MdmX heterodimers. Interestingly, we also identified two mutants that were able to degrade MdmX, 471-472AG, and 473-474AS (Figure 1C), but could not efficiently target p53 for degradation. This result suggested that residues 471–474 could specifically be required for the E3 activity of Mdm2 homodimer and p53 degradation (Figure 6C).

**Figure 7:**
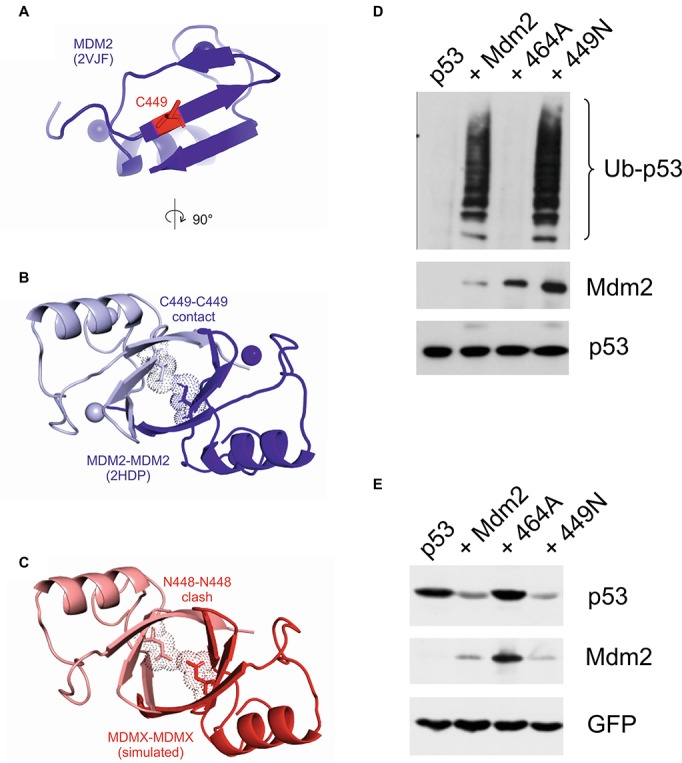
Mutations of the conserved C449 residue can have a different impact on Mdm2 activity toward different targets. **(A)** The Mdm2 dimerization interface (2VJF) (blue) with highlighted central C449 residue (red stick). **(B)** The Mdm2-Mdm2 homodimer (2HDP) with highlighted central C449-C449 contact (sticks and dots). **(C)** In the simulated MdmX-MdmX homodimer (red), the central N448 residues (sticks and dots) are clashing at the dimer interface. **(D)** Cell-based p53 ubiquitylation assay. U2OS cells were transfected with plasmids encoding HA-tagged ubiquitin, FLAG-tagged p53, and wild-type Mdm2 or selected Mdm2 mutants. Cells were treated with a proteasome inhibitor 24 h post-transfection, lysed under denaturing conditions to disrupt non-covalent protein–protein interactions, lysates were diluted, and p53 was immunoprecipitated. Samples were resolved by SDS-PAGE and Western blotting was used to determine the levels of p53 and Mdm2 in cell lysates and HA-ubiquitin linked to p53 in immunoprecipitates. **(E)** p53 degradation assay. U2OS cells were transfected with GFP, FLAG-tagged p53, and wild-type Mdm2 or selected Mdm2 mutants. Cells were lysed 30 h post-transfection and proteins were resolved by SDS-PAGE and analyzed by Western blotting.

We also modeled the RING-RING interface and mapped the position of the critical cysteine 449 and asparagine 448 residues. Two cysteine residues could serve as contact sites at the interface of the Mdm2 RING homodimer (Figures 7A,B). In contrast, in a simulated MdmX RING homodimer, the two asparagine residues clashed and did not seem compatible with homodimerization (Figure 7C). The apparent structural similarity of the RING domains of Mdm2 and MdmX suggested that replacing C449 of Mdm2 with asparagine would disrupt not only its interaction with MdmX but also the homodimerization of the Mdm2 RING and its activity toward p53. Surprisingly, the 449N mutant was active in a p53 ubiquitylation assay (Figure 7D) and capable of inducing p53 degradation (Figure 7E), strongly suggesting that the replacement of C449 with asparagine did not disrupt Mdm2 RING homodimerization. These results indicated that the impact of the two critical residues C449 and N448 on RING dimerization could be context-specific and modified by other RING domain residues.

## Discussion

The complex and dynamic relationship between Mdm2 and MdmX, which is key to the regulation of p53 stability and function, is enabled, at least in part, by the differential ability of the Mdm2 and MdmX RING domains to form dimers and serve as E3 ubiquitin ligases. Although the conserved RING domains of the two proteins exhibit a high degree of homology and structural similarity (Kostic et al., 2006; Linke et al., 2008; Tan et al., 2016), only Mdm2 RING can form homodimers and serve as E3 on its own, while the MdmX RING domain does not appear to have appreciable E3 ubiquitin ligase activity. On the other hand, the MdmX RING domain can actively contribute to Mdm2 E3 activity in the form of the Mdm2-MdmX heterodimer (Kawai et al., 2007; Poyurovsky et al., 2007; Singh et al., 2007; Uldrijan et al., 2007).

The previously published structural analyses indicated possible molecular determinants of the difference in E3 activity between the Mdm2 and MdmX RINGs. Linke et al. have predicted the putative E2-binding site on the Mdm2 RING domain by comparison with the E2/E3 complex of UbcH7 and the RING domain of c-Cbl, and mapped the residues required for functional interaction with the E2 enzyme UbcH5b by mutating the predicted residues to alanine (Linke et al., 2008). Hydrophobic contacts appeared critical since mutations of I440, L468, and P476 abolished activity. Adjacent residues were also deemed important since a mutation of R479 disrupted activity, while mutations of V439 and R471 reduced E3 activity. Many of the surface-exposed residues within Mdm2 predicted to be required for recruiting the E2 are conserved in MdmX, while some of the surrounding residues differ. To determine if these differences render MdmX inactive, Linke et al. mutated selected residues in MdmX to their Mdm2 equivalents, but none of these changes were able to turn on the E3 activity in the MdmX RING domain. The authors concluded that many small differences might contribute to the inactivity of MdmX (Linke et al., 2008).

A recent analysis of the Mdm2RING-MdmXRING-E2(UbcH5b)-ubiquitin complex suggested that despite their apparent structural similarity, the Mdm2 homodimer and Mdm2-MdmX heterodimer have differing abilities to transfer Ub due to their abilities to interact with E2~Ub complexes (~ indicates thioester bond). While Mdm2 RING could bind both UbcH5b and ubiquitin, MdmX did not bind the E2 enzyme and contacted only ubiquitin, *via* its C-terminal tail. The study also identified the conserved arginine residue at position 479 as critical for stabilizing the closed conformation of E2~Ub and the 479K mutant was shown to be defective in discharging UbcH5b~Ub (Nomura et al., 2017).

In the present study, we used PCR-mediated site-directed mutagenesis of the Mdm2 RING domain in the context of full-length Mdm2 to identify residues that could be responsible for the apparent lack of the capacity to homodimerize and low intrinsic E3 activity of the MdmX RING. We mutated parts of the Mdm2 RING into the corresponding MdmX residues and observed a significant drop in the activity toward MdmX in mutants affecting E436, VI439–440, QG442–443, C449, T463, and RQP479–481. Of these mutants, 436K, 439–440SL, 442-442EK, and 479–481KKE could be inactive because they lost at least one residue predicted to participate in the interaction with E2. Surprisingly, however, mutants 439–440SL and 442-442EK were still capable of targeting p53 for degradation, indicating that they remained active in the form of the Mdm2-Mdm2 homodimer. Such a result might suggest that a different set of residues at the Mdm2/E2 interface might be required for E2 binding in Mdm2 homodimers and Mdm2-MdmX heterodimers. Importantly, our mutant 479–481KKE was inactive both in the form of heterodimer and homodimer, confirming the critical contribution of R479 to the ubiquitin ligase activity of Mdm2 (Nomura et al., 2017).

Our study also identified the conserved cysteine 449 of Mdm2 and asparagine 448 of MdmX located at the RING-RING interface as critical determinants of the ability of Mdm proteins to form dimers *in vivo*. When cysteine was introduced into the corresponding position in the MdmX RING, the mutant MdmX protein gained the ability to form homodimers. Our data are consistent with earlier studies in which the RING domain of MdmX was mutated to contain selected Mdm2 residues. Iyappan et al. turned MdmX into an active ubiquitin ligase by mutating N448 to cysteine and replacing residues 465–480 with the corresponding Mdm2 sequence (Iyappan et al., 2010). Egorova and Sheng performed mutational analysis of isolated MdmX RING domain and tested the ubiquitin ligase activity of the mutants *in vitro*. They found that substitution of N448 for cysteine and K478 for arginine granted the MdmX RING domain the E3 activity. Based on subsequent structural analysis, the authors reasoned that C449 of Mdm2 is critical for the stability of the RING dimer structure, while R479 could play a role in recruiting and activating the ubiquitin E2 conjugating enzyme (Egorova and Sheng, 2014). The critical role of cysteine 449 and arginine 479 in the E3 activity of Mdm2 RING was also confirmed by Nomura et al., who found that substituting N448 and K478 to cysteine and arginine, respectively, was sufficient to unmask the ubiquitin ligase activity toward p53 in the Mdm2/X chimera (Nomura et al., 2017).

Interestingly, we found that only one RING with the N448C exchange was required for MdmX RING homodimerization *in vivo*, in line with the results of structural analyses indicating that cysteine and asparagine residues contact in the Mdm2-MdmX heterodimer, but two asparagine residues would clash, preventing MdmX homodimerization. However, in immunoprecipitations, we could detect MdmX homodimerization only when N448 was mutated to cysteine in both MdmX RING domains, suggesting that the context of the RING domain also plays a role. Moreover, the interaction between Mdm2 and MdmX RING domains also seemed unstable when the two critical residues were swapped, as we could not detect the binding of Mdm2 449N to MdmX 448C in the immunoprecipitation assay, despite the predicted compatibility of the two residues. Last but not least, the Mdm2 449N mutant still served as E3 ubiquitin ligase targeting p53 for degradation, again suggesting that the asparagine and cysteine residues had a differential impact on Mdm RING domain function depending on the structural context.

It seems that the tight mutual regulation of Mdm2, MdmX, and p53 activity in cells is enabled by many individual differences between the RINGs of Mdm2 and MdmX that participate in the control of RING dimerization and E2 binding.

## Author Contributions

PK, IS, and AV performed experiments and prepared the figures. BV, PM, and JP analyzed the results and prepared the figures. SU conceived the study, performed experiments, analyzed the results, and wrote the manuscript.

### Conflict of Interest Statement

The authors declare that the research was conducted in the absence of any commercial or financial relationships that could be construed as a potential conflict of interest.
